# Functional Analysis of IL-6 Genetic Variants and Their Potential Role in Lipid Homeostasis and Inflammatory Regulation in Colombian Athletes

**DOI:** 10.3390/cimb48070686

**Published:** 2026-07-03

**Authors:** Diana Carolina Zambrano Ríos, Miguel Ángel Gómez, Juan Manuel Gómez, Felipe Alberto Polo, Betty Oviedo Sarria, Julián Andrés Rivera, Andrés Jenuer Matta

**Affiliations:** 1 Center for Basic Sciences, Escuela Nacional del Deporte, Cali 760001, Colombia; 2 Faculty of Education and Sport Science, Escuela Nacional del Deporte, Cali 760001, Colombia; 3 Faculty of Basic Sciences, Universidad Santiago de Cali, Cali 760001, Colombia

**Keywords:** obesity, interleukin-6, polymorphism, genetic, sports medicine, exome sequencing, Next-Generation Sequencing

## Abstract

Obesity and metabolic dysregulation are closely associated with chronic low-grade inflammation, in which interleukin-6 (IL-6) plays a key regulatory role. Genetic variation in the IL-6 gene may influence inflammatory responses and metabolic homeostasis. To identify single-nucleotide variants (SNVs) in the *IL-6* gene in a cohort of Colombian high-performance athletes and to evaluate their potential functional and structural consequences using bioinformatic prediction and protein-modeling approaches. A descriptive observational study was conducted in a cohort of 23 high-performance Colombian athletes from Valle del Cauca representing cycling, karate, and weightlifting disciplines. Genomic Deoxyribonucleic Acid (DNA) extracted from peripheral blood samples was analyzed using Next-Generation Sequencing (NGS). Identified variants were evaluated using several in silico prediction tools, including Basic Local Alignment Search Tool (BLAST version 2.16.0), Expert Protein Analysis System (ExPASy version 4.0), Open Reading Frame Finder (ORFfinder version 0.4.3), and population databases such as Genome Aggregation Database (gnomAD version 4.0). Structural modeling was used to explore the potential impact of amino-acid substitutions on IL-6 protein stability. Eight single-nucleotide variants were identified in the IL-6 gene. Among them, the rs1524107 variant generated a missense substitution predicted to modify the amino-acid sequence of the IL-6 protein. Structural modeling suggested a potential alteration in protein stability associated with this variant. The rs1524107 variant may influence IL-6 protein structure according to computational predictions. These findings provide preliminary hypothesis-generating evidence regarding the potential role of *IL-6* genetic variation in inflammatory regulation; however, functional validation and larger cohort studies are required to determine their biological significance.

## 1. Introduction

Obesity arises from an excessive accumulation of fat in adipocytes, which increase in size and number, resulting in elevated body mass. This varies among individuals and social groups and represents a significant global issue, having reached pandemic proportions. Obesity is driven by a combination of physiological, psychological, metabolic, genetic, socioeconomic, and cultural factors [1].

According to the World Health Organization (WHO), in 2022, 2.5 billion adults aged 18 and older were overweight, with over 890 million classified as obese [2]. The United States leads in obesity prevalence, with more than 77 million Americans having a body mass index (BMI) of 30 or higher [3]. China and India follow, although their figures are proportionally lower despite their large populations [4]. Colombia is among the top 100 countries with the highest obesity rates; however, it has the lowest levels of obesity in Latin America [5]. Nonetheless, exposure to risk factors for obesity has increased among the population.

Obesity is associated with multiple hormonal, inflammatory, and endothelial alterations that contribute to the development of several chronic diseases, including type 2 diabetes mellitus (T2DM), hepatic steatosis, cardiovascular disease, stroke, dyslipidemia, hypertension, osteoarthritis, sleep apnea, and certain types of cancer [6]. 

Individuals with obesity commonly present a chronic low-grade inflammatory state associated with triglyceride accumulation and adipose tissue expansion. Adipose tissue contains fibroblasts, preadipocytes, adipocytes, macrophages, and multiple components of the innate immune system.

The innate immune system constitutes the first line of defense against microorganisms and tissue injury. Its activation is mediated through receptors encoded in the germ line, among which Toll-like receptors (TLRs) are the most extensively studied. Activation of these receptors promotes the production of inflammatory mediators, including tumor necrosis factor alpha (TNF-α), interleukin-6 (IL-6), and interleukin-1 beta (IL-1β).

Interleukin-6 (IL-6) regulates anti-inflammatory cytokines such as Interleukin-1 receptor antagonist (IL-1Ra) and Interleukin-10 (IL-10) and influences carbohydrate and lipid metabolism [7]. Consequently, IL-6 is associated with obesity, inflammation, and lipid metabolic pathways. This study aimed to identify genetic variants in the *IL-6* gene in a cohort of high-performance Colombian athletes from Valle del Cauca and to explore their potential functional relevance through bioinformatic prediction and structural modeling approaches within the context of inflammatory and metabolic regulation.

## 2. Materials and Methods

### 2.1. Type of Study and Population

This exploratory observational study was conducted in a cohort of 23 high-performance athletes from Valle del Cauca, Colombia, between August 2022 and August 2023. Participants represented three competitive sports disciplines: cycling (*n* = 8), karate (*n* = 3), and weightlifting (*n* = 12). The mean age of the participants was 20 years (range: 18–25 years). All the participants were evaluated for the presence of polymorphisms in the IL-6 gene using NGS.

The inclusion criteria were as follows: 1. being an athlete officially registered with the Municipal Institute of Sports and Recreation of Valle del Cauca (IMDERVALLE); 2. having obtained medals at the local, national, and/or international level on more than three occasions, indicating a high level of sports performance; 3. absence of diagnosed genetic diseases in family members up to the third degree of consanguinity; and 4. voluntary agreement to participate in the study through reading, understanding, and signing the informed consent form.

The exclusion criteria included: 1. voluntary withdrawal from the study by the athlete and 2. failure to attend scheduled evaluations or interviews required for data collection.

After agreeing to participate in the study, all the individuals read and signed an informed consent form that explained the study objectives, sampling procedures, and potential risks associated with participation. All the collected information and results were subsequently recorded in a database specifically designed for this research.

### 2.2. Ethical Considerations

All the participants received detailed information regarding the objectives, procedures, potential risks, and benefits of the study and provided written informed consent prior to enrollment. The study was conducted in accordance with the ethical principles established in the Declaration of Helsinki and complied with Colombian regulations governing research involving human participants, including Resolution 8430 of 1993 issued by the Ministry of Health of Colombia.

The study protocol was reviewed and approved by the Human Research Ethics Committee of the Institución Universitaria Escuela Nacional del Deporte under Resolution No. 40.07.232.

### 2.3. DNA Extraction, Library Preparation and Sequencing

Peripheral venous blood samples were collected from all the participants using sterile EDTA-containing tubes. Genomic DNA was isolated from whole blood using the QIAamp DNA Blood Mini Kit (Qiagen^®^, Hilden, Germany) according to the manufacturer’s instructions. DNA quality and integrity were evaluated by amplification of the *glyceraldehyde-3-phosphate dehydrogenase* (*GAPDH*) gene and spectrophotometric quantification using a NanoDrop One spectrophotometer (Thermo Fisher Scientific, Wilmington, DE, USA).

Sequencing libraries were prepared following the protocols established by the sequencing laboratory. NGS was performed to identify genetic variants within the *IL-6* gene, sequencing quality control procedures were applied throughout the workflow, and an average sequencing depth of approximately 100× was achieved.

Raw sequence reads were aligned against the *Homo sapiens* reference genome GRCh38 (Genome Reference Consortium Human Build 38), and variant calling was performed using the bioinformatic pipeline implemented by the sequencing provider. All the identified variants were subsequently annotated and evaluated through computational analyses to determine their potential biological relevance. 

### 2.4. Bioinformatic Analysis

Bioinformatic analyses were conducted to characterize the identified *IL-6* variants and to explore their potential biological significance. In silico methods refer to computational approaches that use bioinformatic algorithms, biological databases, and predictive models to evaluate genetic variation and generate hypotheses regarding potential functional effects. These methods are widely used in genomic research for variant prioritization and functional prediction but do not constitute direct experimental validation.

Sequence alignment and variant verification were performed using the Basic Local Alignment Search Tool (BLAST version 2.16.0), which identifies regions of local similarity between nucleotide sequences and facilitates sequence annotation; open reading frames were evaluated using ORFfinder version 4.0, while protein sequence analyses and molecular characterization were supported by the ExPASy version 4.0 bioinformatics resource portal.

Population allele frequencies were investigated using the gnomAD version 4.0, a large-scale reference resource containing genomic information from diverse populations worldwide. Functional relationships among genes were explored using GeneMANIA version 3.6.0, which integrates data on co-expression, co-localization, genetic interactions, pathways, and physical interactions to predict gene function and biological networks.

Protein–protein interaction networks were evaluated using STRING version 12.0 (Search Tool for the Retrieval of Interacting Genes/Proteins), a platform that integrates experimental evidence, curated databases, and computational predictions to identify functional associations among proteins. These analyses were used to explore the biological context of IL-6 and its interaction with proteins involved in inflammatory and metabolic pathways. 

Finally, the detected variants were categorized according to their genomic position, nucleotide substitution, amino-acid change, variant classification (synonymous, missense, or intronic), and population allele frequency. All the identified variants were compared with reference sequences available in the National Center for Biotechnology Information (NCBI) databases to ensure accurate annotation. In addition, the potential biological relevance of each variant was evaluated considering both the predicted structural effects on the protein and the distribution of allele frequencies in population databases.

### 2.5. Bioinformatic Tools and In Silico Functional Prediction

In silico methods refer to computational approaches used to analyze biological data and predict the potential functional consequences of genetic variation through bioinformatic algorithms, sequence analysis, structural modeling, and database annotation. These methodologies are widely used in genomic research to prioritize variants of potential biological relevance and generate hypotheses for subsequent experimental validation.

Sequence alignment and variant verification were performed using the BLAST version 2.16.0, a computational algorithm developed by the NCBI that identifies regions of local similarity between biological sequences and facilitates sequence annotation and variant confirmation, ORF identification was performed using ORFfinder version 4.0, while protein sequence analyses were supported by the ExPASy version 4.0 bioinformatics resource portal [8].

Population allele frequencies were evaluated using the gnomAD version 4.0, a large-scale reference database containing genomic data from diverse populations that allows assessment of variant prevalence and population distribution [9].

Functional relationships among genes were explored using GeneMANIA version 3.6.0, a web-based prediction platform that integrates multiple biological datasets, including co-expression, physical interactions, pathways, co-localization, and genetic interactions, to identify functionally related genes and biological networks [10].

Protein–protein interaction networks were investigated using STRING, a database that integrates experimental evidence, computational predictions, and curated biological information to characterize potential functional interactions among proteins [11].

The potential functional consequences of genetic variants were further evaluated using Combined Annotation Dependent Depletion (CADD version v1.6), which integrates multiple genomic annotations into a single deleteriousness score, and SpliceAI, a deep learning-based algorithm designed to predict the impact of genetic variants on RNA splicing [12].

Structural analyses were performed using Swiss-PdbViewer version 4.1.0, a molecular visualization and protein modeling platform that allows the evaluation of potential structural alterations associated with amino-acid substitutions. The results generated by these computational tools were interpreted as predictive evidence only and do not constitute direct functional validation [13].

### 2.6. Statistical Analysis

Given the exploratory nature of the study and the limited sample size (*n* = 23), no inferential statistical analyses or genotype–phenotype association tests were performed. The study was designed primarily to identify genetic variants within the *IL-6* gene and to evaluate their potential functional relevance through computational prediction approaches.

Descriptive statistics were used to summarize the distribution and frequency of the identified variants. Allele frequencies observed in the study cohort were compared descriptively with population frequencies reported in the gnomAD version 4.0. No statistical comparisons between groups were conducted, and therefore, the findings should be interpreted as exploratory and hypothesis-generating rather than confirmatory.

Finally, the demographic and sports characteristics of the participants were summarized using descriptive statistics. Continuous variables were expressed as mean ± standard deviation and range, whereas categorical variables were reported as absolute frequencies and relative percentages; descriptive analyses were performed for the overall cohort and according to sport discipline. All the statistical calculations were performed using Python software (version 3.11) with the pandas library (version 2.0).

## 3. Results

The study included 23 high-performance Colombian athletes from three sport disciplines: weightlifting (52.2%), cycling (34.8%), and karate (13.0%). The population was predominantly male (65.2%), with an overall mean age of 23.2 ± 3.0 years. Detailed sociodemographic characteristics of the participants are presented in Table 1.

Eight single-nucleotide variant (SNV) polymorphisms with the highest allele frequencies in the *IL-6* gene were identified among the 23 participants. Among these, rs1524107 is a missense heterozygous mutation, while rs155460, an intronic variant, is the least frequent according to the gnomAD (Table 2).

To evaluate the potential effects of *IL-6* variants on protein structure and function, bioinformatic analyses were performed using BLAST, ExPASy, and NCBI ORFfinder. These analyses suggested that rs1524107 may influence the amino-acid sequence and potentially affect the structural stability of the IL-6 protein (Table 3).

Table 2 also presents the CADD and SpliceAI scores for the identified variants. The CADD values represent which polymorphisms have an effect on the functionality of the protein [14], and among them, we have the rs1524107, while with SpliceAI, it was observed that all the polymorphisms identified modify the splicing sites with values < 0.5, which increases the probability of modifying the structure and functioning of the protein.

All the observed polymorphisms were evaluated with the Swiss-PdbViewer application, which is a tool for the study of proteins, their molecular modeling, protein engineering, homology studies, prediction of folding or three-dimensional structure (through threading) and analysis of simulation results and molecular dynamics [15,16], and it was obtained that rs1524107 modified the stability of the protein by reducing the Gibbs free energy.

**Figure 1:**
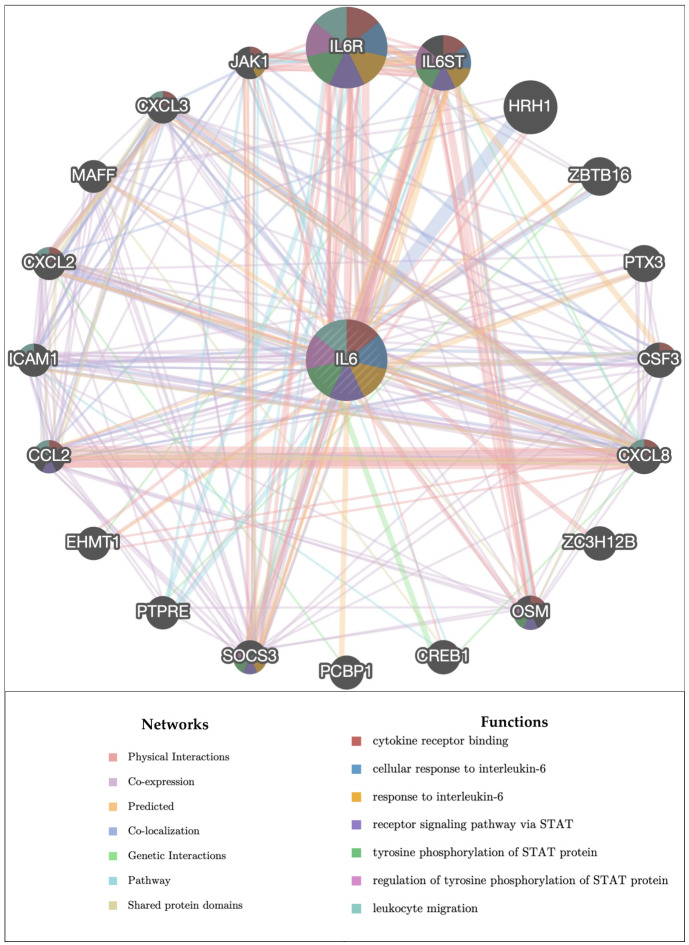
IL-6 gene and its co-expression with other genes (taken from geneMANIA). The network diagram was generated using the GeneMANIA platform (version 3.6.0) and is reproduced under a Creative Commons Attribution 4.0 International (CC BY 4.0) license.

With the geneMANIA results, we observed that the *IL-6* gene is related to genes that encode proteins that are inflammatory biomarkers *Interleukin-6 receptor* (*IL-6R*), *Interleukin-6 signal transducer/gp130* (*IL-6ST*), *Oncostatin M* (*OSM*) [17,18,19,20] and participate in angiogenesis, tissue renewal, induce the differentiation, mobilization and growth of neutrophils *IL-6R*, *C-X-C motif chemokine ligand 2/MIP-2-alpha* (*CXCL-2*), *C-X-C motif chemokine ligand 3/GRO-gamma* (*CXCL-3*), *C-C motif chemokine ligand 2/MCP-1* (*CCL2*), and *Interleukin-8/IL-8* (*CXCL-8*) [18,19,21,22], which are important processes in athlete recovery, Figure 1.

**Figure 2:**
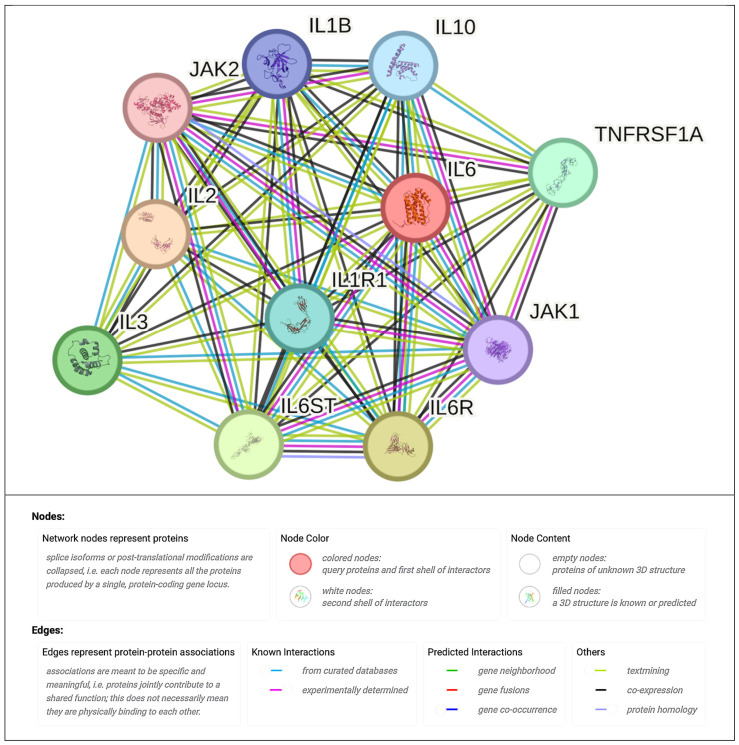
IL-6 and its co-expression with proteins (taken from String). The network image was generated using the STRING database (version 12.0) and is reproduced under a Creative Commons Attribution 4.0 International (CC BY 4.0) license.

With the STRING software, the interaction between the IL-6 protein and others within the cell signaling pathways was evaluated Figure 2, and the SpliceAI analysis showed scores below 0.5 for all the identified variants, suggesting a low predicted impact on splicing sites [23,24,25,26,27,28].

Given the exploratory nature of the study and the limited number of participants, no statistical genotype–phenotype association analyses were performed. Instead, the study focused on the identification and in silico functional prioritization of *IL-6* genetic variants.

## 4. Discussion

The present study identified several SNVs within the *IL-6* gene in a cohort of high-performance Colombian athletes using NGS. Among the detected variants, rs1524107 was prioritized for further analysis because computational prediction tools suggested a potential impact on protein structure and stability.

Interleukin-6 is a multifunctional cytokine involved in immune regulation, inflammatory responses, and metabolic adaptation. Elevated circulating IL-6 concentrations have been associated with obesity, visceral adiposity, and chronic low-grade inflammation, highlighting its relevance in both physiological and pathological processes [16,28]. In addition, IL-6 plays a central role in host defense mechanisms and immune regulation through interactions with multiple cellular and molecular pathways [28,29].

Among the variants identified in the present study, rs1524107 was prioritized for structural analysis based on its predicted functional annotation. Computational analyses performed using BLAST, ExPASy, ORFfinder, CADD, SpliceAI, and Swiss-PdbViewer suggested that this variant may influence structural properties of the IL-6 protein. Structural modeling indicated a potential alteration in protein stability and conformation; however, these observations should be interpreted with caution because they are derived exclusively from bioinformatic prediction tools and do not constitute direct evidence of altered protein function.

Computational prediction approaches have become valuable resources for prioritizing variants that may warrant further biological investigation; CADD integrates multiple genomic annotations to estimate the potential deleteriousness of genetic variants, whereas structural modeling tools provide information regarding possible effects on protein architecture. Nevertheless, these methodologies generate predictive evidence rather than experimentally validated functional evidence and therefore should be considered hypothesis-generating.

The network analyses performed using GeneMANIA and STRING demonstrated that *IL-6* is functionally connected with genes and proteins involved in inflammatory regulation, immune-cell recruitment, angiogenesis, tissue remodeling, and cellular homeostasis, among these interactions were molecules associated with leukocyte adhesion and migration, such as Intercellular Adhesion Molecule 1 (ICAM-1) [17], chemokines including CXCL8 and CXCL2 [19,20], and proteins involved in neutrophil mobilization and tissue repair [18,23], additional interactions were observed with cytokine-related pathways involving OSM, a member of the IL-6 cytokine family with recognized immunomodulatory functions [22].

The interaction networks identified in the present study are consistent with previous reports demonstrating the participation of IL-6 in exercise-induced immune responses and inflammatory regulation; acute and chronic exercise are known to influence cytokine production and signaling, including IL-6-mediated responses that contribute to metabolic adaptation, immune surveillance, and tissue recovery [24,30,31,32]. Furthermore, IL-6 has been reported to influence lipid metabolism and energy regulation in skeletal muscle, reinforcing its relevance in athletic populations [21,30].

The STRING analysis also highlighted interactions between IL-6 and proteins involved in inflammatory signaling pathways. Previous studies have demonstrated that IL-6 signaling is mediated through highly regulated receptor systems and intracellular cascades, including the Janus Kinase/Signal Transducer and Activator of Transcription (JAK–STAT) pathway [29,33,34]; however, although structural modeling suggested that rs1524107 may influence IL-6 protein conformation, the present study did not experimentally evaluate receptor binding, signal transduction efficiency, or downstream pathway activation. Consequently, no conclusions can be drawn regarding the potential effects of this variant on IL-6 receptor interactions or intracellular signaling mechanisms.

Previous investigations have suggested that genetic variation in cytokine-related genes may contribute to inter-individual differences in immune responses, inflammatory regulation, and adaptation to physiological stress [32]. In this context, the identification of IL-6 variants in athletes provides preliminary information that may support future studies exploring the relationship between genetic variation and exercise-related physiological responses.

The findings should therefore be interpreted within the exploratory scope of the study. Given the limited sample size and the absence of phenotypic, metabolic, inflammatory, or functional measurements, no genotype–phenotype associations could be established. Future investigations involving larger cohorts, comprehensive phenotypic characterization, and experimental validation will be necessary to determine whether the identified variants exert measurable biological effects and to clarify their physiological and clinical relevance.

## 5. Conclusions

The evidence presented highlights the biological relevance of IL-6 as an important mediator linking inflammation, energy metabolism, and immune regulation. IL-6 has been widely described as a cytokine involved in both pro-inflammatory responses associated with adipose tissue expansion and metabolic regulation during skeletal muscle activity.

In the present study, several single-nucleotide variants were identified in the *IL-6* gene in a group of Colombian athletes using Next-Generation Sequencing. Among these variants, rs1524107 generated a missense substitution that, according to in silico structural modeling, may potentially influence the amino acid sequence and structural stability of the IL-6 protein.

Computational analyses further suggested that the rs1524107 variant could influence IL-6 protein conformation and potentially affect receptor interaction or downstream signaling pathways. However, these observations are based exclusively on predictive bioinformatic tools, including structural modeling and functional annotation platforms, which are valuable for hypothesis generation but do not constitute direct functional evidence.

Accordingly, experimental studies will be required to determine whether this variant exerts measurable effects on IL-6/IL-6R signaling or downstream pathways such as JAK–STAT 3 or AMP-activated Protein Kinase (AMPK). Therefore, no definitive conclusions regarding pathway disruption can be established from the present data. This distinction ensures that the conclusions remain consistent with the methodological scope of the present study, which was focused on variant identification and computational functional prediction.

Additionally, the present study did not include phenotypic or metabolic measurements that would allow direct genotype–phenotype correlation. Consequently, no association can be established between rs1524107 and metabolic or inflammatory outcomes in this cohort. Future studies involving larger populations and incorporating metabolic, inflammatory, and performance-related phenotypes will be necessary to clarify the biological and clinical significance of *IL-6* genetic variants.

Overall, these findings provide preliminary and exploratory evidence that genetic variation within the IL-6 gene may contribute to inter-individual variability in inflammatory regulation and metabolic pathways. Nevertheless, further experimental validation and integrative genotype–phenotype analyses are required to confirm the functional and physiological relevance of the identified variants.

## 6. Limitations

This study has several limitations that should be considered when interpreting the findings. First, the relatively small sample size (*n* = 23) limits the statistical power and generalizability of the results. Due to the exploratory nature of the study and the limited number of participants, no genotype–phenotype association analyses were performed.

Second, the study relied primarily on in silico bioinformatic and structural prediction tools, including sequence annotation, functional prediction, and protein modeling approaches. Although these computational methods are valuable for variant prioritization and hypothesis generation, they do not constitute direct functional or experimental evidence; therefore, the predicted effects of the identified variants on IL-6 structure or signaling pathways should be interpreted with caution.

Third, no phenotypic, metabolic, inflammatory, or clinical measurements were collected from participants; consequently, direct associations between the identified IL-6 variants and obesity-related phenotypes, inflammatory status, metabolic alterations, or athletic performance could not be established.

Additionally, some variants identified in this study were in intronic or non-coding regions, making their biological significance difficult to determine without complementary functional assays. Future studies involving larger cohorts, comprehensive phenotypic characterization, transcriptomic analyses, and experimental validation will be necessary to clarify the biological and clinical relevance of *IL-6* genetic variation.

## Figures and Tables

**Table 1:** Sociodemographic characteristics of the study population.

Sport Discipline	*n* (%)	Sex		Mean Age ± SD
		Female *n* (%)	Male *n* (%)	
Weightlifting	12 (52.2%)	5 (41.6%)	7 (58.3%)	21.8 ± 2.7
Cycling	8 (34.8%)	3 (37.5%)	5 (62.5%)	23.0 ± 2.3
Karate	3 (13.0%)	0 (0%)	3 (100%)	25.0 ± 6.1
Total/Overall	23 (100%)	8 (34.8%)	15 (65.2%)	23.2 ± 3.0

**Table 2:** Observed variants of the IL-6 gene and their frequency in the population studied.

Position	Nucleotide Change	Type of Variation	NCBI Reference	Variant Effect	Allele	Allele Frequency genomAD	Allele Frequency. (*n* = 23)
22,728,600	c.211-93C>T	SNV	rs1524107	Missense	Heterozygote	0.0802	12
22,728,630	c.211-63G>T	SNV	rs2066992	Intron	Homozygote	0.0801	12
22,727,814	c.210+180A>G	SNV	rs2069832	Intron	Homozygote	0.9269	17
22,730,530	c.*744G>A	SNV	rs2069845	3’ UTR	Homozygote	0.6167	17
22,729,088	c.324+282T>G	SNV	rs1554606	Intron	Homozygote	0.6634	18
22,728,289	c.211-404C>G	SNV	rs1474348	Intron	Homozygote	0.9271	21
22,728,045	c.210+411C>T	SNV	rs1554299912	Intron	Homozygote	0.9272	20
22,728,505	c.211-188C>A	SNV	rs1474347	Intron	Homozygote	0.8437	22

* CADD: Combined Annotation Dependent Depletion. SpliceAI: A deep learning-based tool to identify splice variants. genomAD: Genome Aggregation Database.

**Table 3:** In silico interpretation of the effect of genetic variation on the amino acid sequence of the IL-6 protein.

NCBI Reference	Nucleotide Change	Reference Amino Acid	Alternating Amino Acid	CADD	SpliceAI
rs1524107	c.211-93C>T	T	V	10.8	0.00
rs2066992	c.211-63G>T	T	V	16.6	0.00
rs2069832	c.210+180A>G	T	V	0.489	0.00
rs2069845	c.*744G>A	S	G	4.25	0.00
rs1554606	c.324+282T>G	T	V	1.73	0.00
rs1474348	c.211-404C>G	T	V	2.62	0.02
rs1554299912	c.210+411C>T	S	G	7.99	0.00
rs1474347	c.211-188C>A	T	V	4,61	0.01

* CADD: Combined Annotation Dependent Depletion. SpliceAI: A deep learning-based tool to identify splice variants. genomAD: Genome Aggregation Database.

## Data Availability

Data availability is subject to authorization from the ethics committee. The project’s principal investigator will escalate the request to the committee and based on the response, share the information in the respective repositories for review.

## Abbreviations

The following abbreviations are used in this manuscript:

BLAST	Basic Local Alignment Search Tool
BMI	Body Mass Index
CAAD	Cancer-Associated Adipocytes
CXCL-2	C-X-C Motif Chemokine Ligand 2
CXCL-3	C-X-C Motif Chemokine Ligand 3
CCL2	C-C Motif Chemokine Ligand 2
CXCL-8	Interleukin-8
DNA	Deoxyribonucleic Acid
GAPDH	Glyceraldehyde-3-Phosphate Dehydrogenase
gnomAD	Genome Aggregation Database
GRCh38	Genome Reference Consortium Human Build 38
IL-6	Interleukin 6
IL-6R	Interleukin-6 Receptor
IL-6ST	Interleukin-6 Signal Transducer
IL-12	Interleukin-12
IL1-β	Interleukin-1 beta
IL-1Ra	Interleukin-1 Receptor Antagonist
NCBI	National Center for Biotechnology Information
NGS	Next-Generation Sequencing
OSM	Oncostatin-M
SNV	Single-Nucleotide Variant
STRING	Search Tool for the Retrieval of Interacting Genes/Proteins
T2DM	Type 2 diabetes mellitus
TLR’s	Toll-Like Receptors
TNF-α	Tumor Necrosis Factor-A
WHO	World Health Organization
